# “You’ve Gotta be Careful”: Familial Messages Regarding Sexual Behavior and Sexual Relationships among African American Adolescents

**DOI:** 10.3390/ijerph16071146

**Published:** 2019-03-30

**Authors:** Gary W. Harper, Darnell N. Motley, April Timmons Tyler, Donald H. Tyler, Joseph A. Catania, M. Margaret Dolcini

**Affiliations:** 1 School of Public Health, University of Michigan, Ann Arbor, MI 48109, USA; 2 Department of Obstetrics & Gynecology, University of Chicago, Chicago, IL 60637, USA; dmotley@bsd.uchicago.edu; 3 Michael Reese Research and Education Foundation Care Program at Mercy Hospital, Chicago, IL 60616, USA; april.timmons@gmail.com; 4 A New Way of Being, LLC, Chicago, IL 60637, USA; anwb.behavioralhealth@gmail.com; 5 Hallie Ford Center for Healthy Children and Families, College of Public Health and Human Sciences, Oregon State University, Corvallis, OR 97331, USA; catania1951@comcast.net (J.A.C.); Peggy.Dolcini@oregonstate.edu (M.M.D.)

**Keywords:** adolescence, African Americans, family systems perspective, qualitative methods

## Abstract

Background: Research on the sexual behaviors of African American youth has primarily focused on associated risks, with a dearth of studies examining a fuller representation of African American adolescents’ sexual lives. This study explored the range of messages African American adolescents receive from family members regarding sexual behavior and sexual relationships. Methods: Participants were 52 sexually experienced African American youth (male = 32, female = 20) between the ages of 15 and 17 recruited from community-based organizations in the United States. Youth participated in individual in-depth qualitative interviews, and data were analyzed using a phenomenological framework. Results: Participants received a variety of messages about sexual behavior and sexual relationships from a range of family members including parents, siblings, grandmothers, aunts/uncles, and cousins. Types of messages clustered into three domains: sexual decision-making, quantity and quality of sexual activity, and sexual health promotion; with themes and sub-themes emerging within each area. Conclusion: Gender differences in the types of messages received are explored, and applications of the findings to the development of family-involved community interventions that promote sexual and reproductive health are discussed.

## 1. Introduction

The bulk of research that has sought to examine the sexual behaviors of African American youth has been focused primarily on associated risks, particularly risks of intimate partner violence [1,2], unplanned pregnancy [3,4], or the transmission of Human Immunodeficiency Virus (HIV) and other sexually transmitted infections (STIs) [5,6,7]. These studies are often in reaction to data demonstrating disproportionately higher rates of some negative sexual health outcomes among African American youth, suggesting the potential presence of culturally specific patterns of sexual behavior as well as culturally specific influences on sexual behavior at multiple socioecological levels. What is often missing are studies that offer a counter-narrative regarding the sexual lives of African American youth, exploring how specific cultural experiences and messages may serve to promote healthy sexuality and to protect African American youth from negative sexual health outcomes. Few studies have sought to develop a complex understanding of African American adolescents’ sexuality, including culturally specific health-promoting aspects of their sexual knowledge and experiences. To gain a complex understanding of the sexual lives of African American youth and the array of sociocultural influences on their sexual behaviors, it is important to consider the range of sexual messages they receive about sex and sexuality.

In addition to research on the sexual lives of African American adolescents, health promoting community interventions are needed to promote the sexual health of these youth and it is important to understand how culture may influence normative sexual development when creating such interventions. Utilizing a cultural framework will allow for the development of community-based interventions that are rooted in the culture of African American adolescents and, as such, may have the potential for more lasting behavioral change. In the present study, particular attention was given to the central role of the extended family in the developmental trajectory of African American adolescents [8,9,10] and the range of messages received from family members regarding sex. This allowed for an assessment of the breadth of messages youth receive from various family members.

### 1.1. The African-Centered Behavioral Change Model

African-centered psychologists have developed culturally-specific frameworks for understanding factors and forces that impact the lived experiences of African American children, adolescents and adults [11,12]. The African-Centered Behavioral Change Model (ACBCM) argues that culture is the central factor that influences the behavior of African American people [13]. In fact, Nobles, Goddard, and Gilbert (2009) referred to this cultural environment as the “culturecology” and argued that the cultural traditions, precepts, norms, values, customs, and beliefs unique to a particular cultural community must be considered and respected in order to initiate behavior change [13].

The ACBCM [13] is, accordingly, grounded in the fundamental notion that ideas, as manifestations of culture, are the substance of behavior and that behavior is the result of the choices made and chances taken. These choices and chances are the product of culturally grounded notions regarding African American humanity and value. The necessity of this focus on humanity and value is predicated on the idea that the historical experience of African Americans has had detrimental effects that are demonstrated in various ways throughout the culture (e.g., media representations of African American people) that inhibit notions of African American value. Given these conditions, the ACBCM argues that a process of “culturalization”, where African American life is affirmed and negative social conditions are no longer a primary focus, is necessary to promote positive behavioral change. This culturalization process is accomplished through aligning oneself with values that affirm the value of African American people and culture and promoting agency as a means for asserting control over one’s life. Similar to other processes of socialization, much of this work is or can be done in the context of family.

### 1.2. The Role of Extended Family in African American Adolescent Development

The familial make-up of African American families runs the gamut, including two-parent homes, households led by a single parent, and more complex arrangements where there might be other relatives that have become a part of the familial unit [10]. Given the growing number of African American children being raised in single mother homes, researchers have often focused on analysis of the parent–child dyad (most often mother–child) without considering the potential role of other family members who assist in the childrearing process [14,15,16]. This is problematic, given that African American children are often raised in close proximity to other relatives who offer support to the parent via childcare and assistance with daily aspects of living such as transportation to work or errands [17,18]. In addition, approximately 40% of African American adults share a residence with a non-partner relative, as compared to less than 20% of White adults [9].

It has been argued that the centrality of the extended family in African American communities is attributable to vestiges of African values and customs, which defined family broadly rather than only including the nuclear family [19,20]. These relatives were not only viewed as proximal, but they were charged with ensuring the health and well-being of one another as well as one another’s children [19,20]. As such, the presence of extended family in the daily lives of modern African American children is in keeping with cultural traditions.

In sum, the presence of extended family members has been demonstrated to have both cultural and practical utility. Accordingly, the prevalence of non-parent adults as additional child caretakers is sufficiently high among African Americans, particularly those with limited financial resources, which necessitates their inclusion in any meaningful analysis of the impact of the family on African American adolescent development.

### 1.3. Family Influences on Sexual Activity

It has been long established that parents play an important role in the sexual socialization of their adolescent children [21,22], with a recent process review reinforcing this parental influence and highlighting the importance of parent–child sex communication to decrease negative sexual health outcomes during adolescence [23]. Previous research has demonstrated that parents and other family members play a central role in the development of African American adolescents’ attitudes regarding sexual behavior [24,25,26]. Several studies and a systematic review have demonstrated that African American adolescents whose parents talk with them about sexual activity and sexual protection are more likely to delay the onset of sexual activity and/or engage in lower rates of sexual risk behaviors than their counterparts whose parents do not discuss such matters [27,28,29]. In addition, parent–adolescent communication has also been found to buffer the effects of peers who may be encouraging youth to have sexual activity [30,31].

Gender differences have been found with regard to the nature of conversations that parents have with their adolescent children. Akers and colleagues (2011) found that there are differences in the focus of sex-relevant conversation depending upon the gender of the youth with whom they are sharing information [32]. When talking to daughters, relationship safety was a primary focus while the primary focus with sons was the sons’ capacity to show respect for partners [32]. Aronowitz, Todd, Agbeshie, and Rennells (2007) found that African American mothers primarily communicated negative messages about males and their sexual intents (e.g., they are “predators” and only want sex; they are not honest) to their daughters [33]. This implies that the quality of the information received by young men and young women may differ, with young men not learning as much about how to protect themselves emotionally in a sexual relationship and young women not receiving information about how to express their sexual desires.

The majority of studies examining familial communication about sex have focused on communication between mothers and daughters [23,29], while less is known about the nature and influence of father–son sexual health communication [34,35]. One recent exception is a qualitative study by Randolph and colleagues (2017), who found that African American fathers perceived that relationship and communication quality would facilitate conversations with their son, while developmental readiness of the child, communication discomfort and not talking to their own fathers about sex when growing up would serve as barriers to such discussions [35]. This study focused on fathers’ perceptions of barriers and facilitators (not necessarily past experiences), and also shared a limitation of other studies in that it did not talk directly to adolescents about their experiences with such conversations.

While an understanding of the role of parents in the development of African American adolescent’s sexual attitudes, values, and behaviors is valuable, given the important position of non-parental family members in the lives of these youth [8], it is important to understand their role in influencing adolescent’s views of sexual relationships and sexual behavior. In two different qualitative studies, Wallace et al. (2012) demonstrated the critical role that siblings play in socializing African American youth regarding sexual relationships, sexual behavior and sexual health [26], while Harper et al. (2012) demonstrated that African American youth receive messages about dating and romantic relationships from a variety of different family members [25]. While some of the messages in this latter study came from parents, they were also received from siblings, aunts, uncles, cousins, grandparents, and even a father’s girlfriend.

The current study focused on exploring the range of messages African American adolescents receive from family members regarding sexual behavior and sexual relationships. Although prior research has examined the influence of parent–adolescent communication on the sexual attitudes and behaviors of adolescents, studies have not conducted an in-depth analysis of the range of messages youth receive from multiple family members regarding sexual behavior and sexual relationships for heterosexual African American adolescents. Given the strong influence of extended family members on African American adolescents [8,9,10], it is critical to understand the potential influences of an array of family members including mothers, fathers, brothers, sisters, aunts, uncles, grandmothers, and grandfathers.

## 2. Materials and Methods

### 2.1. Participants

Participants for this study were 52 African American youth (male = 32, female = 20) between ages 15 and 17 at recruitment. These youth represented a subsample of adolescents who participated in a larger research study focused on gender ideologies and their relationship to dating and sex. To take part in the study, participants had to have met the following criteria: (1) identify as African American or Black; (2) be between ages 15 and 17 (inclusive); and (3) endorse predominant sexual behavior with persons of the other sex. Since the dating and sexual behaviors of adolescents whose predominant sexual attractions and/or sexual experiences are with members of the same sex (regardless of their sexual orientation identity) will likely differ from those whose predominant sexual attractions and sexual experiences are with members of the opposite sex, the parent study centered specifically on this latter group. The 52 youth selected for analysis in this study met the criteria for the larger study but additionally met the criteria of being sexually active, as defined by having ever had penetrative intercourse with a person of the other sex.

### 2.2. Procedure

All participants were recruited from community-based, youth-serving agencies in either Chicago or San Francisco, United States. In each city, the research team worked with one primary community agency that was a major provider of youth-focused programs for African American youth in their geographic area, and these agencies worked collaboratively with other community agencies as well. Youth who participated were living in low-income neighborhoods within these major United States cities. The neighborhoods from which the participants were selected were comparable with respect to high rates of poverty, unemployment, and STI prevalence [36,37,38].

The Internal Review Boards of the participating academic institutions approved this study. A trained member of the research team established eligibility for the larger study through a screening procedure, and then obtained parental consent and youth assent. The youth then participated in individual, semi-structured qualitative interviews administered by trained interviewers in a private room located in the designated primary community agency within each city. When possible, the gender of the participant and interviewer were matched.

All interviews took place in Spring and Summer 2010, and participants were compensated for their time. Interviews were digitally recorded and transcribed. Accuracy of transcription was checked by members of the research team involved in data collection.

### 2.3. Interview Guide

A semi-structured qualitative interview guide was developed specifically for the larger study by a team of researchers experienced in working with African American youth. The interview was grounded in a phenomenological framework, which provided a general structure for discussion but required participants to provide their own definitions based on life experiences and perceptions. The interview protocol covered multiple aspects of sex and dating relationships, including gendered images of African American adolescents, sources of information about sex, norms about sexual activity and relationships, advice about dating and relationships, and sexual communication. In line with the phenomenological framework, the term “family” was not specifically defined in the interview, so youth were allowed to identify and discuss any individuals who they viewed as “family”.

### 2.4. Data Analysis

Data analysis was conducted using a phenomenological framework as well, focusing the analysis on describing what a given group of participants have in common as they experience a particular phenomenon [39,40]. To assist with classifying, sorting, and retrieving coded text during the analysis process, transcribed interviews were entered into QSR International’s NVivo 7 software (Melbourne, Australia) prior to analysis.

Data coding and analysis were iterative and interactive processes conducted by a team of four qualitative analysts, all of whom had clinical experience working with African American youth and three of whom identify as African American. The first step involved reading all interview transcripts to increase familiarity with the data. After all of the transcripts were read and reviewed, content codes were created to capture the experiences described by participants, and a codebook was created that included operational definitions of all codes. Transcripts were then reread and pattern codes were created to connect subsequent concepts under larger headings within each transcript. Following this, consistent patterns in meaning, concepts, and themes across all interviews were identified [39,41]. Data matrices were created as visual representations of the findings to further assist in the analysis process, including comparative matrices that identified the gender of the youth who received each type of message and the specific sources of each message.

The team of analysts discussed coding and analysis activities during research meetings, and discrepancies in coding and interpretation were resolved through discussion and consensus. Given the study’s phenomenological framework, all themes and sub-themes expressed by participants, regardless of their frequency of occurrence, were considered meaningful elements of how each particular phenomenon was experienced by the participant(s) and thus included in the analysis.

## 3. Results

The results below demonstrate the variety of messages African American youth receive about sexual behavior and sexual relationships from their family members. These messages regarding sexual and reproductive behaviors are organized into three domains: sexual decision-making, quantity and quality of sexual activity, and sexual health promotion. Sexual decision-making messages described factors which should determine how and with whom a youth should engage in sexual behavior. Quantity and quality of sexual activity messages described the number of sexual partners a youth should have as well as specific characteristics of the sexual activity/relationships they should have. Sexual health promotion messages described ways that the youths could protect their sexual heath.

Within each domain of messages, salient themes and sub-themes which emerged from the data are presented accompanied by one or more illustrative quotations. Several quotations contain the letter “R” at the beginning of a statement to denote that it is the respondent speaking, or a letter “I” at the beginning of a statement to denote that it is the interviewer speaking. Pseudonyms have been used to ensure the confidentiality of participants.

### 3.1. Sexual Decision-Making

Within the domain of sexual decision-making messages, three primary themes emerged. These included selection of sexual partners, potential impacts of sexual involvement, and how to avoid sexual activity.

Messages related to selection of sexual partners described the kinds of sexual partners that would be preferable for a youth. Some of these messages urged youth to, in general, be selective when choosing a potential sexual partner.

*I: Has [your dad] shared anything that was helpful?*

*R: About having sex? Don’t have sex with everything and anything.*

*I: So if he said don’t have sex with anything and everything, who’s he saying have sex with? Did he share who you should have sex with?*

*R: No. Just said choose the right person basically. Or wait.*

*I: Did he describe to you what that right person is?*

*R: Nah. (Sam, 15-year-old male youth)*

*I: Did you hear any advice from [your mom] about boyfriends and sex?*

*R: Yeah. She told me to be careful, and don’t just do it to anybody. And be a wise consumer. (Sandy, 15-year-old female youth)*

In addition to these general partner selection messages, some youth also received specific messages about what sort of sexual partner would or would not be preferable. When asked about messages he had received about sex, Jabari, a 16-year-old young man, reported being advised by his mother to avoid “stunts”, promiscuous girls who may have a high number of sexual partners.

*[My mom] don’t want me to bring a stunt home to her and say this is my girl; she want me to bring someone classy or someone that didn’t have a lot of sex. (Jabari, 16-year-old male youth)*

Messages regarding potential impacts of sexual involvement described a number of potential outcomes. Some messages described potential negative impacts on academic achievement, but the majority of these messages focused on the potential for pregnancy and STI transmission. The permanence of these outcomes was often stressed, as demonstrated by 16-year-old Marie’s response.

*You don’t know what could happen. You could get pregnant, [my mom] says, you don’t want to have no kids and you don’t want to be walking around with no STD or HIV for the rest of your life. (Marie, 16-year-old female youth)*

While most of these messages were meant to caution youth about their sexual activity, there were also relatives who encouraged youth to have sex due to particular considerations. For example, Rebecca’s mother and cousin encouraged her to have sex with a young man she was dating in order to avoid his breaking up with her.

*I: Have you ever gotten advice on boyfriends and sex?*

*R: Um-hum. I talk to my cousin about sex, my mom about sex.*

*I: What kind of advice do they give you?*

*R: Only advice that I got, if you don’t do it, another girl will. Like you know, so if your boy want you to do it, try it at least once. Like you know, or he’ll find another girl who will do it. Things like that. (Rebecca, 16-year-old female youth)*

The last theme within the domain of sexual decision-making messages was messages about how to avoid sexual activity if not interested. Family members offered a variety of strategies to the youth, which clustered into the following avoidance sub-themes: direct avoidance strategies, indirect avoidance strategies, ways to take control of sexual decision-making, and utilizing familial advice.

Direct avoidance strategies described activities the youth could do to avoid having sex when not interested. Many family members encouraged youth to just say “no”, and youth were also encouraged to resist peer pressure no matter what.

*I: What about from family members, do they ever share anything about ways to avoid having sex?*

*R: Only thing my grandma and my mom and my sister always tell me don’t let no boy put nothing in your head. If you don’t want to open up your legs to that boy, say no. That’s basically my grandma used to always say. My mom used to say, don’t ever let no boy peer pressure you into having sex if you don’t want to. (Kayla, 15 year old female youth)*

Indirect avoidance strategies described by family members were related to avoiding behaviors which might suggest sexual desire or interest. Sometimes these behaviors were ones that more obviously communicated sexual interest or availability, but other times, the sexual implication was less clear. For example, Danielle, a 16-year-old young woman, was advised to avoid talking on the phone to a young man after a certain time of day.

*I know one of my cousins, she older, like she older, older, and she – I think she almost 60. She always be sayin’ like if I’m on the phone and I’m around her and it’s like getting close to night time she got in her mind, “Shouldn’t be on the phone with that boy. It’s late, ‘cause he might think something.” (Danielle, 16-year- old female youth)*

When encouraging youth to take control of sexual decision-making, family members focused on reinforcing the youth’s agency. While somewhat akin to the encouragement youth received related to avoiding unwanted sexual contact, these messages were more general and did not encourage or discourage sexual involvement. These messages instead focused on the existence of an option and supporting the youth’s knowledge that it was his/her choice to make.

*R: Like everybody tells me like, “It’s your decision where – if you want to have sex or not to have sex.” Like that’s what I’ve been hearing, and that’s what I’ve been told and everything. That’s why I just follow off that, like if I want to or not.*

*I: Okay. Who has told you this—that it’s your decision?*

*R: Mostly everybody. Family and friends. Family. Friends.*

*I: Which family members?*

*R: Most all of ‘em. (Ken, 17-year-old male youth)*

Last, family members encouraged youth to seek additional counsel from them if they later have more specific questions or concerns about sexual involvement. This would allow them to respond to specific situations in the youth’s life and support him/her in making the best decision. It could also facilitate the family member assisting in providing necessary supports (e.g., prophylactics) to the youth. One young woman’s mother even stated that it would allow her to bring in additional persons if the mother’s knowledge was insufficient.

*“If you ever feel like you’re pressured to have sex and you think you want to have sex with some boy,” [participant’s mother] said, “I mean I can try my hardest to convince you not to have sex but at the end of the day that’s something that you want to do it’s something you want to do, just come talk to me so I can put you on birth control. And so I can talk to you and get someone else to talk to you about all the risks there is.” (Nia, 16-year-old female youth)*

### 3.2. Quantity and Quality of Sexual Activity

Within the domain of quantity and quality of sexual activity messages, two primary themes emerged: quantity and quality. Messages about the quantity of sexual activity described how many persons a youth should be involved with sexually. In general, there was a great deal of agreement when such messages were given. Family members discouraged youth from having too many partners and some encouraged youth specifically to only have one partner.

*My mom would rather me have sex with one person than two or three or ten or nine. (Amy, 15-year-old female youth)*

*R: It’s nasty to have too many partners.*

*I: From who did you get that idea?*

*R: The women in the – my family, like my grandmother and my mom, auntie and stuff like that. (Rob, 17-year -old male youth)*

Messages about the quality of sexual activity clustered into two sub-themes: descriptions of the physical act of sex and normative sexual behaviors.

In describing the physical act of sex, family members generally spoke to young women about the potential pain related to sexual activity. Kristi, a 17-year-old young woman, described how her sister described the pain of sexual intercourse, but also made clear that she did not entertain the communication much.

*R: Then [my sister will] mention—she like, “Yeah, it really hurts.” I’m like, “I’ve actually heard that, but okay.”*

*I: Wait. It hurts when you…*

*R: When you first have sex or something like that. She’s like, “It really hurts,” and I’ll be like, “Okay. Bye. I don’t care. Go away,” and then she’ll keep talking about it. (Kristi, 17-year-old female youth)*

Donna, another 17-year-old young woman, described her family giving her information about how to engage in sexual intercourse. However, her family members also described pain in relation to sex, which inhibited her interest in participating in sexual activity.

*R: And like I know when we was growing up – like when they started having sex, they told me. Like they told me like – because I already knew what it was and stuff like that. So they just told me like, “This is what to do.” Like all this little stuff. And just broke it down for me to learn.*

*And I was just like, “Okay. I understand. I’m not having sex.” Because they just scared me to not have it.*

*I: So what did they say?*

*R: They were just like, “You know it hurts. You’re gonna bleed.” Stuff like that. And just scared me. (Donna, 17-year-old female youth)*

Messages about normative sexual behaviors described a baseline of what the youth were to understand as being appropriate and “normal” sexual acts. These messages described both acts that should be understood as normative and acts that should be understood as non-normative, strange, or taboo. Across multiple participants, particular taboo was attributed to anal sex, as demonstrated by 15-year-old Frederick’s response.

*I used to think anal sex was, like, normal sex, but I learned [from my brother] that ain’t where it’s supposed to go. (Frederick, 15-year-old male youth)*

### 3.3. Sexual Health Promotion

Within the domain of sexual health promotion, three primary themes emerged. These included messages about pregnancy prevention behaviors, STI prevention behaviors, and general sexual health maintenance behaviors. This final domain represented the most robust area about which family members provided messages to youth.

Many of the youth described family members encouraging them to practice behaviors which could inhibit risks of pregnancy. Many youth reported receiving general information about birth control and condoms. However, some other youth reported more specific messages about how to effectively communicate about birth control and condoms and how to effectively use birth control and condoms.

General information about birth control provided by family members was related to how to use birth control and condoms, the diversity of birth control options, and potential side effects to birth control use. These messages were all couched in or following larger messages about the utility of birth control and condoms for avoiding pregnancy.

*I: What did [your brother] say?*

*R: No, he—I don’t know, he just always told me to use a condom. My mom always—my dad almost always told me that, too.*

*I: Okay, and why did he say use a condom?*

*R: Because I’m too young to have babies.*

*I: Um-hum, okay. Have you asked another person for advice on girlfriends?*

*R: Not really though, it just be my brother and my dad. They be telling me stuff. My brother mostly though, he just be telling me stuff like, “Just use condoms.” He been telling me to use condoms. (Ray, 17-year-old male youth)*

*I: Okay. And what did you learn from family?*

*R: Same thing. You can get all different types, different ones. But they told me that some birth control gives you different—like reactions. Like some people gain weight. Some lose weight. Some people, I don’t know. (Portia, 15-year-old female youth)*

While the majority of the messages about female birth control methods were to young women, young men also received messages about these birth control methods as well.

*I: So tell me what you learned from your mom and from your dad.*

*R: She told me that [birth control] affects a girl’s body. It changes her hormones and stuff like that. My dad just told me that your girl can’t get or there’s more of a chance that she won’t get pregnant on birth control. (Chris, 16-year-old male youth)*

In addition to information about the utility and function of birth control and condoms, youth also received messages from their family members about how to discuss these things with a sexual partner.

*I talked to my boyfriend and I learned to talk to him from my mom like how to start it, like oh, so babe, I was thinking about using birth control. What do you think about that? Like not making like, oh, I’m going to get on birth control and you gonna like it. Like you know? You got to see how they feel and if they don’t like it, just go do it and don’t go in the bed. (Rebecca, 16-year-old female youth)*

While some messages related to condom use were related specifically to the prevention of pregnancy, the bulk of messages related to condom use were related to the prevention of STI transmission.

*I: Okay. What did you learn from home?*

*R: To definitely use one [condom], so you don’t have no babies – or no disease or nothing like that.*

*I: Now who—?*

*R: My mother and my sister. (Darrell, 17-year-old male youth)*

*R: And like my grandma—she always used to tell me if I’m going to have sex, wear a condom. But she just don’t want me to have sex. And my friends—they always talk about condoms. I mean some of them got pregnant, so that’s why they tell us.*

*Like me and my best friend just lost our virginity. So they tell us like, “You guys wear a condom. Don’t do it without a condom.” You know?*

*I: Don’t do it, to prevent pregnancy?*

*R: Yeah. And like they’re like, “Y’all got to make sure he’s only having sex with you. Because he can get an STD and stuff like that.” And all the little stuff like that. But yeah. (Josie, 17-year-old female youth)*

In addition to the messages family members gave youth about using birth control and condoms to avoid pregnancy and STI transmission, youth also received more general messages about maintaining their sexual health through hygiene and regular medical care.

*I: So from [your cousins, sisters, and aunties], what did you learn about how a girl should carry herself?*

*R: They should carry their self respectable, and basically like a young lady.*

*I: What does that mean – carry yourself like a lady?*

*R: Like hygiene stuff, and keep yourself up… If you have sex, get on birth control. Use douches. Go to the doctor. You and your sex partner get a checkup, and stuff like that. And use condoms. (Christina, 15-year-old female youth)*

Shelby, a 16-year-old woman, described an experience where her father was so preoccupied with the idea that she was sexually active that he was not responding to her immediate sexual health needs. In this situation, her father’s then-girlfriend intervened and supported her seeking necessarily medical services.

*And I remember one time, I asked my dad, can I go to my appointment. He was like, “You havin’ sex?” He already knew that I was though, but he just wanted to see what I was gonna say, and I’m just sittin’ there, and I’m like, “So can I go to my appointment?” And he like, “XXX, you havin’ sex? Is there something you not tellin’ me? You think you got something? Do I need to take you down there?” And then my dad’s girlfriend just snatched the phone. Her name, XX [dad’s ex-girlfriend], the one that taught me everything, she like, “If you need to go to the clinic go, just go, okay?” She like, “Don’t trip off your dad. You dad a tripper right now. But if there’s anything you gotta tell me, just know you can talk to me, okay?” I’m like, “All right.” She like, “All right.” And I just went. (Shelby, 16-year-old female youth)*

### 3.4. Types and Sources of Messages

We explored the gender of the youth who received the messages revealed in the phenomenological analysis, as well as the specific family members who shared those messages. The source of the message was classified according to how the youth described the person from whom they received the specific type of message. For these sources of the message, we categorized them as either coming from parental figures and siblings, or from other family members. These data are presented in Table 1.

Messages about sex were received from a wide array of different types of family members including parents/parental figures (mother, father, and father’s girlfriend), siblings (sister and brother), grandmothers, aunts/uncles, and cousins. No messages were reported as being transmitted by grandfathers, and some youth talked about receiving specific messages from family members but did not specify the type of family member who shared that view (designated as “unspecified family member” in Table 1). Three (33.3%) of the nine different types of messages were only received by one gender, and these were all transmitted to female youth and not male youth: potential impacts of sexual involvement, describing the sexual act, and gender sexual health maintenance behaviors. For these three messages, they were conveyed to female youth only by female family members (with the possible exception of one family member’s whose specific identity category was not revealed). The other six messages were shared with both female and male youth and were transmitted to the youth from a variety of both male and female family members.

Further examination of the sources of the messages revealed that the most common source of the nine different types of messages was mothers or maternal figures (7 of 9; 77.8%), followed by sisters (5 of 9; 55.5%), fathers (4 of 9; 44.4%), cousins (4 of 9; 44.4%), and grandmothers (3 of 9; 33.3%). Other family members were mentioned as sources for two or less types of messages. The only two types of messages that were not conveyed by mothers/maternal figures were those related to messages about specific sexual practices or behaviors, including describing the physical act of sex and normative sexual behaviors—these were only expressed by sisters or unspecified family members.

## 4. Discussion

### 4.1. Messages in the Context of ACBCM

Youth in this study received a variety of messages from family members regarding sex, and these messages were transmitted by an array of different family members. The most common source of the nine different types of messages conveyed to youth was mothers or maternal figures (77.8% of message types), followed by sisters (55.5%), fathers (44.4%), cousins (44.4%), and grandmothers (33.3%). The greater frequency of messages by mothers supports prior research focused on mother-child communication processes for African American youth [14,15,16], but the frequency of messages by sisters, fathers, cousins, and grandmothers suggests a need for more research on the role of non-maternal family members in conveying information about sexual behavior and sexual relationships.

The majority of the messages received by participants were protective, intending to give youth advice that would improve their sexual experiences, including the selection of sexual partners, the implications of being sexually involved, and the healthiness of their sexual practices. In addition, many of the messages given to these youth by their family members were intended to socialize them into community norms regarding sex (e.g., the negative value associated with anal sex). While this is also arguably a protective measure intended to ensure their ability to function in their cultural context, it also demonstrates the role of this extended family network as co-rearers of these youth. In addition, the only two types of messages that were not conveyed by maternal figures were those about specific sexual behaviors or activities, with these types of messages being conveyed by sisters or unspecified family members. This family-member-specific transmission of different types of sexual behavior/sexual relationship messages also supports the notion of extended family networks contributing to the development and socialization of African American youth.

Many of the messages these youth received were similar across genders. For example, both genders were encouraged to have relatively few partners and to seek sexual partners who have a similarly low number of sexual partners. However, sometimes traditional gender ideologies were manifested in both the general category of messages that were received, as well as the specific type of information that was provided in the message. Three of the nine general categories of messages were only received by female youth. Most of these messages were cautionary or protective in nature, related to potential negative consequences of having sex (e.g., STIs, pregnancy, poor academic achievement), benefits of maintaining sexual health (both hygiene and sexual medical care), and concerns regarding the pain of sexual activity—while one female-specific message was related to using sex as a way to maintain a boyfriend. Although both female and male youth received messages about pregnancy and STI prevention, young women were advised about both condoms and birth control, whereas messages for young men focused solely on condoms, as if birth control were not in their purview.

These gender differences in the messages that family members share with their children have been found in prior studies as well [32,33]. The cautionary nature of the female-specific messages found in the current study was also revealed by Aker et al. (2011) and Aronowitz et al. (2007), both of whom demonstrated that parents were more likely to warn their daughters about the dangers of sexuality and sexual relationships than their sons [32,33]. Although Aker et al. (2011) found that parents shared gender-specific messages with their sons about respecting their female sexual and romantic partners [32], the current study did not reveal any male-specific messaging regarding sexuality other than a greater focus on condoms over other forms of birth control.

Despite how gender ideologies may have limited the scope of messages that youth of each gender received, youth of both genders were consistently receiving messages of some sort that intended to ensure that their sexual relationships, whenever they should begin, would be ones that were absent of negative consequences. The consistent existence of these messages highlights the fact that family members are engaging youth in discussions about sex and sexual health. This is emblematic of the culturization process described by the ACBCM model [13], as it focuses on the value of these youth’s lives and stresses the health-positive choices these youth have available to them. Despite these youths living in low-income neighborhoods impacted by multiple stressors, the messages offered by their family members focus on the youth’s inherent potential and the necessity of preserving it.

The network of relatives from whom youth received messages included persons with a range of ages, from older siblings and cousins to parents, aunts, uncles, and grandparents. This demonstrates a wide co-rearing network with a diversity of experiences invested in protecting the health and well-being of these youth through an intergenerational transmission of knowledge. This supports prior research that demonstrates the central role of the extended family in the developmental trajectory of African American adolescents [8,9,10]. Having such a diverse and extensive network in the form of family messaging and support related to sexual and reproductive health may help to promote the resilience of these youth as they are exposed to the stressors associated with living in low-income environments. Such resources, coupled with more individual-level assets, may disrupt the relationship between social and environmental risk factors and negative sexual health outcomes such as pregnancy, STIs, and HIV [42,43].

The current study expands the extant literature on the role that family members play in the development of adolescents’ knowledge, attitudes, and beliefs about sexual activity and sexual health [22,25], and adds critical information about the types and sources of messages youth receive from family members about sexual activity. It also offers a different perspective on the concept of parent–child communication among African American youth, since the majority of existing studies have focused on the sexual health-related benefits of parents engaging in specific conversations with their children about sexual activity and sexual protection. Data from the current study were focused on naturally occurring familial messages and conversations between youth and their family members, as opposed to more specific and deliberate conversations, which offers another perspective on how parents may be influencing their children’s sexuality.

### 4.2. Implications for Community Interventions

The array of health-promoting messages that African American youth are receiving from their family members suggests that family members should be actively engaged in community interventions geared toward impacting the sexual health and wellness behaviors of these young people. This may include facilitating continued or increased communication between youth and their family members, but it may also include providing simultaneous sexual health education to family members and youth. While the messages family members offered to youth were generally intended to protect them and promote their health and wellbeing, not all messages were fully accurate. As such, engaging youth and their family members with simultaneous health education serves to ensure that all parties have the most accurate information.

Coordinated interventions with parents and their children may also give an opportunity for a normalizing of communication about areas that were previously considered taboo. For example, the familial rejection of anal sex as non-normative may limit youth’s understanding of how to safely engage in anal sex, should they decide to do so. Interventionists would be able to educate both the family members and the youth about safe anal sex practices, thereby informing all parties but also potentially removing taboo around discussion of the practice. A further benefit of engaging various family members of youth about sexual health is that education conferred to them could positively affect other youth not directly involved in the intervention when these family members share advice about how to engage in sexual relationships.

### 4.3. Strengths, Limitations, and Future Research

This study sought to systematically examine the breadth of messages African American youth receive from their family networks regarding sexual behaviors and relationships. Given the role of this extended family network in the development of African American youth, this analysis is critical. Further, the sample for this study included a large number of participants for a qualitative study of this type (*n* = 51) from two urban cities representing both the West Coast and Midwest regions of the United States, and focused on youth who were early in their sexual lives (ages 15–17). This is a critical time in the development of adolescents’ norms around sexual behaviors and relationships, as they are beginning to determine their attitudes, norms, and behavior patterns related to sex. Accordingly, understanding the diversity of messages they are receiving at this time informs what sort of community interventions may be best in order to strengthen these youth’s knowledge of health-positive sexual options.

Despite these strengths, there were limitations to this study. Participants were recruited from community-based organizations in low-income communities; therefore, it is likely that only youth from lower socioeconomic status families were included. In addition, these youth also may represent more highly motivated youth since they were attending (and many receiving services from) community agencies. Since the focus of the larger study from which these data were extracted was not specifically on messages youth received from family members regarding sexual relationships, the interviews may not have offered the level of depth that would reveal a more nuanced and detailed understanding of these messages. In addition, information regarding sources of the various messages was not rigorously assessed during the interview since this was not a focus of the study. In addition, since the interviews were conducted at only one point in time, it was not possible to explore the ultimate impact of familial messages on attitudes, norms and behaviors related to sexual relationships. Finally, the sample only included those youth who reported predominant sexual activity with members of the opposite sex, thus the findings may not have as much relevance for those youth whose predominant sexual attractions and/or sexual experiences are with members of the same sex. Sexual minority adolescents may have an array of different experiences related to same-sex attraction, dating and sexual behavior, as well as a range of family messages about both same-sex and opposite-sex sexual behavior and relationships, thus tailored research with this particular population is warranted.

Future research regarding familial messages transmitted to African American youth would benefit from sampling youth from various geographic regions of the United States to see if geographic differences are present in the types and sources of messages received. Such studies also may benefit from longitudinal data collection in order to more accurately assess how these messages may change over time, and also how these messages may impact changes in behavior. In addition, more in-depth and nuanced data collection regarding the various sources of these messages would be quite useful, as well as more complex analyses of the types of messages conveyed by these various family sources. Sampling older adolescents also may reveal any age-specific messaging that may occur, and including same-sex attracted youth (both those who identify with a sexual minority sexual orientation identity and those who do not) may offer insights into potential differences in the messages they receive about dating and sexual relationships. Including interviews with various family members would also be beneficial, since data in the current study relied exclusively on self-reports from the perspective of the youth. Youth may have selectively remembered either specific types of messages received, or messages received from specific family members. Mixed-methods (qualitative and quantitative) data collection also has the potential to enhance our understanding of familial messages received by African American youth, and could provide a more nuanced view of both the messages received and the influence of these messages on sexual behavior.

## 5. Conclusions

This study provides some of the first in-depth qualitative data on the range of messages heterosexual African American youth in the United States receive from multiple family members regarding sexual behavior and sexual relationships. These messages are organized into three domains: sexual decision-making, quantity and quality of sexual activity, and sexual health promotion. Sexual decision-making messages described factors that should determine how and with whom a youth should engage in sexual behavior. Quantity and quality of sexual activity messages described the number of sexual partners a youth should have as well as specific characteristics of the sexual activity/relationships they should have. Sexual health promotion messages described ways that the youths could protect their sexual heath.

The majority of these messages were intended to give youth advice that would improve their sexual experiences, including the selection of sexual partners, the implications of being sexually involved, and the healthiness of their sexual practices. In addition, many of the messages were intended to socialize youth into community norms regarding sex. The network of relatives from whom youth received messages included persons with a range of ages, from older siblings and cousins to parents, aunts, uncles, and grandparents. These family members, diverse in ages and life experiences, were all invested in protecting the health and well-being of African American youth through an intergenerational transmission of knowledge. Our findings support prior research that demonstrates the central role of the extended family in the developmental trajectory of African American adolescents, and suggests that future sexual and reproductive health interventions for this population will benefit from the inclusion and active engagement of a range of family members.

## Figures and Tables

**Table 1:** Types and Sources of Message Received about Sex and Sexual Activity.

Type of Message	Gender of Youth Receiving Message	Sources of Message: Parental Figures and Siblings	Sources of Messages: Other Family Members
Selection of sexual partners	Female, Male	Mother, Father, Sister	Cousin
Potential impacts of sexual involvement	Female	Mother	Unspecified Family Member
How to avoid sexual activity	Female, Male	Mother, Sister	Grandmother, Cousin, Unspecified Family Member
Quantity of sexual activity	Female, Male	Mother, Father	Grandmother, Aunt, Uncle
Describing the physical act of sex	Female	Sister	Unspecified Family Member
Normative sexual behaviors	Female, Male	-	Unspecified Family Member
Pregnancy prevention behaviors	Female, Male	Mother, Father, Brother	Cousin, Unspecified Family Member
STI prevention behaviors	Female, Male	Mother, Sister, Brother	Grandmother
General sexual health maintenance behaviors	Female	Father’s Girlfriend, Sister	Aunt, Cousin
